# The role of the two splice variants and extranuclear pathway on Ki-67 regulation in non-cancer and cancer cells

**DOI:** 10.1371/journal.pone.0171815

**Published:** 2017-02-10

**Authors:** Luca Chierico, Loris Rizzello, Lijuan Guan, Adrian Steve Joseph, Andrew Lewis, Giuseppe Battaglia

**Affiliations:** 1 Department of Chemistry, University College London, London, United Kingdom; 2 Biocompatibles UK Ltd., Farnham Business Park, Weydon Lane, Farnham, United Kingdom; Children's Hospital Boston, UNITED STATES

## Abstract

Ki-67 is a nuclear protein that has been used in cancer diagnostic because of its specific cell-cycle dependent expression profile. After quantifying and characterising the expression level of Ki-67, as a function of the cell cycle, we found out that the two main splice variants of the protein (*i*.*e*. α and β) are differently regulated in non-cancerous and cancerous cells both at mRNA and protein level. We were able to correlate the presence of the α variant of the protein with the progression through the interphase of cell cycle. We also observed that the different expression profiles correspond to different degradation pathways for non-cancerous and cancerous cells. Furthermore, Ki-67 is continuously regulated and degraded via proteasome system in both cell types, suggesting an active control of the protein. However we also observed a putative extranuclear elimination pathway of Ki-67 where it is transported to the Golgi apparatus. Our evidence in the different expression of the splice variants may represent a milestone for the development of new targets for cancer diagnostic and prognostic. Additionally, the unexpected extranuclear elimination of Ki-67 strongly suggests that this protein must be looked at also outside of the “nuclear box”, as thought to date.

## Introduction

Ki-67 is a nuclear protein, encoded by the gene MKI67 on the chromosome 10 (10q26), which is present in two main different splice variants of 320 and 359 kDa [1]. It has been demonstrated to play a pivotal role in cell proliferation, as well as it is associated with rRNA transcription [2, 3]. The expression of Ki-67 is strongly promoted during the phases G_1_, S, and G_2_ of the cell cycle (namely, the active states), while the protein, and its codifying mRNA, are both rapidly down-expressed when cells exit the active cell cycle (*i*.*e*., the G_0_) [2, 4, 5]. This cell transit, via mitosis, is promoted by the cyclin B/cdc2-dependet phospho- and Ki-67 dephosphorylation pathways [6]. The exclusive expression in specific phases of the cell cycle, combined with an overall short half-life, made the protein an ideal candidate for the development of specific antibodies useful for quantifying the cell proliferation, a crucial topic in cancer research [7–9]. It should be mentioned that several studies highlighted Ki-67 as a strong prognostic biomarker for both breast and non-small cell lung cancer [3, 10]. This, in turn, has important implications for the further selection and improvement of follow-up protocols/treatments based on radiation, chemotherapy, and/or surgery.

Only very recently the bio-molecular function of Ki-67 has been finally uncovered [11]. In particular, Ki-67 acts as surfactants and prevents chromosomes from collapsing into chromatin immediately after the disassembly of the nuclear membrane during cell division. This allows correct chromosome motility and an effective interaction with the mitotic spindle [11]. Despite these promising data, there are however still a high level of uncertainty, especially regarding the exact function of each Ki-67 splice variant (the α and the β). Many efforts have been mainly focused toward the exploration of Ki-67 as a specific structural nuclear element [4, 5, 12–15]. However, there are not specific studies available on potential extra-nuclear Ki-67 pathways, in order to better understand its possible fate beyond the nucleus as well. In addition, we still miss the bigger picture on how the two splice variants are regulated, especially in different cells.

In this work, we explored new avenues in Ki-67 regulation in cancerous and non-cancerous cells, especially in the viewpoint of addressing a relation between the proliferation activity of cells and the splice variants expressed, as well as with the protein degradation pathway.

We first studied the expression profile and cellular localisation of Ki-67 in different cells, namely in non-cancerous primary human dermal fibroblasts (HDF) and human umbilical vein endothelial cells (HUVEC), as well as in human breast cancer cells (MDA-MB-231), human cervix adenocarcinoma cells (HeLa), and hypopharyngeal carcinoma cells (FaDu). This preliminary investigation enabled us to understand how different cells regulate the protein as a function of the different steps of the interphase. Then, we focused on the possible relationship between the two main splice variants of Ki-67 and the cellular control over proliferation. Together with the control over the transcriptional regulation of the specific variants, imaging investigations further confirmed that cells control the post-translational expression of Ki-67 via proteasome degradation.

Finally, quantitative imaging confocal assays surprisingly suggest that Ki-67 may be translocated outside of the nucleus in non-cancerous cells, resulting in accumulation within the endoplasmic reticulum first, and the Golgi apparatus later. This transfer mechanism seemed significantly less efficient in cancerous cells. This would support the idea that extranuclear effectors may finely regulate the degradation of Ki-67, and that the transfer between the different stages of the interphase of cell cycle is associated with the cytosolic fate of the protein, and with the specific splice variant present.

## Results

### Quantification of Ki-67 expression in different cell models

**Fig 1:**
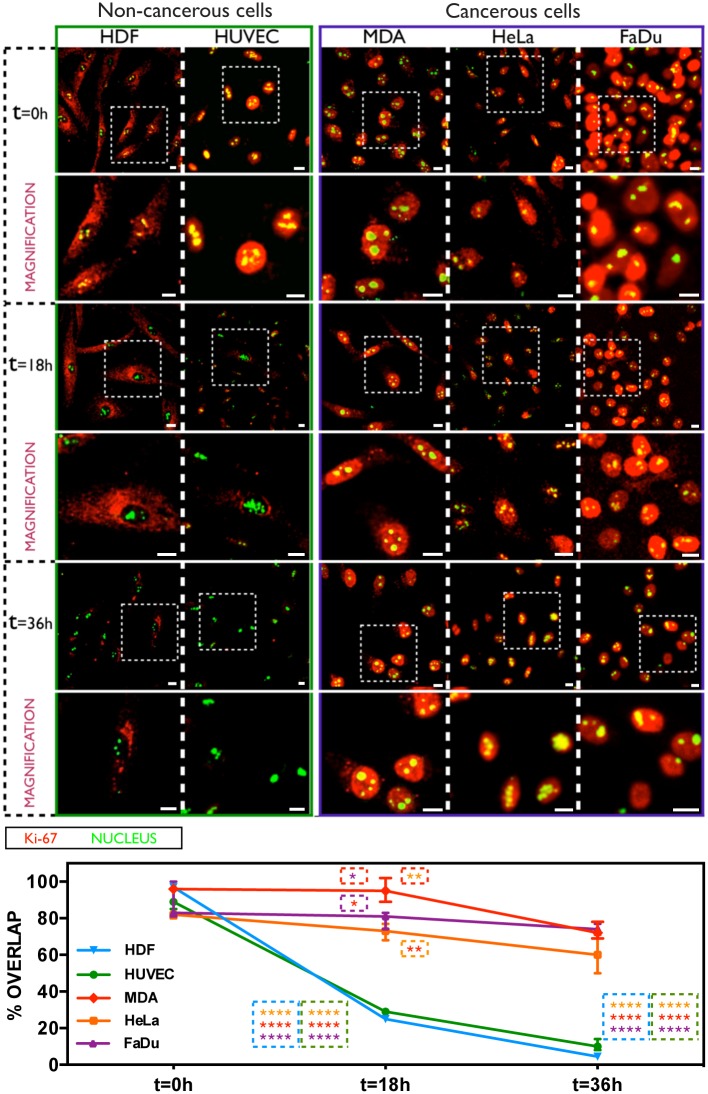
Ki-67 expression in non-cancerous and cancerous cells upon serum starvation. Confocal analysis of Ki-67 expression in HDF, HUVEC, MDA-MB-231, HeLa and FaDu cells (analysed over time after serum deprivation), and the relative fluorescence signal quantification (average overlap value percent). (Two-way ANOVA, p-value * P ≤ 0.05; ** P ≤ 0.01; *** P ≤ 0.001; **** P ≤ 0.0001). Scale bar: 10 μm.

The first experimental approach aimed to quantify and localise the intracellular Ki-67 in non-cancerous and cancerous cells, as a function of their proliferative or quiescent phases. Immunofluorescence investigations were carried out on HDF and HUVEC (non-cancerous cells), as well as on MDA-MB-231, FaDu, and HeLa cells (cancerous cells), co-labelling them with fluorescent anti-Ki-67 antibodies and with SYTO^®^9 for nuclear staining. Cells were incubated in both normal and Fetal Bovine Serum (FBS) deprived media to control the proliferation status. We found high levels of Ki-67 at nuclear level immediately after starvation (time zero) of primary HDF and HUVEC cells (Fig 1, top-left). However, the amount of protein becomes significant less after 18 hours of starvation time, and it is completely undetected after 36 hours of FBS deprivation, when cells enter the quiescent phase. On the other hand, Ki-67 is always present even after 36 hours of starvation time in cancer MDA-MB-231, HeLa, and FaDu cells (Fig 1). A starvation time-dependent quantification of Ki-67 confirmed a strong fluorescence decrease for HDF and HUVEC cells, which shifts down of ∼70% after 18 hours of starvation, and becomes *c*.*a*. 80% and 98% less after 36 hours for HUVEC and HDF, respectively (bottom part of Fig 1). This condition is significantly different in cancerous cells undergoing serum starvation, where a down-expression of Ki-67 was not detected. The quantification of the protein signal is, in fact, roughly the same between 0 and 18 hours of FBS deprivation, and slightly decreases of ∼25% after 36 hours of starvation time (Fig 1).

We then studied the progression through the different steps of the interphase (*i*.*e*., the G_0_-to-G_2_ phases) as a function of the Ki-67 expression in HDF and MDA-MB-231 cells (considered, for the next experimental approaches, as representative models for non-cancerous and cancerous cells, respectively).

In particular, propidium iodide (PI) based fluorescence cytometry (FC) analyses confirmed that ~62% of HDF cells, incubated with complete growth medium, are in the G_0_/G_1_ phase (they are quiescent/synchronised), and ~38% are in the S/G_2_/M steps (*i*.*e*., in a proliferative status), as shown in the left side of Figure A in S1 File. Upon 36 hours of serum starvation, ~98% of fibroblasts are in the G_0_/G_1_, and only 2% of cells are in active S/G_2_/M phases (left section of Figure A-A in S1 File). On the other hand, a significant difference between normal cultured and starved MDA-MB-231 cells is not evident. FC analyses confirmed that ~54% of breast cancer cells in complete medium are in the G_0_/G_1_ stage, and ~46% lies in the S/G_2_/M. Similarly, ~75% of MDA-MB-231 cells stack in the G_0_/G_1_, and ~25% of cells are in the S/G_2_/M upon starvation (right side of Figure A-A in S1 File).

This first evidence confirms the inability of breast cancer cells to control the interphase check points (even after serum deprivation), and enabled to set the nutrient conditions for the next experimental approach, where the presence of Ki-67 has been further quantified as a function of the interphase sub-phases. In particular, HDF cultured in normal medium displayed the presence of Ki-67, while the protein is strongly down regulated during the G_0_/G_1_ stationary phase (Figure A-B of S1 File). This is evident by the strong overlap between the blue and grey graph of Figure A-B of S1 File (left side), and by the relative quantification signal. On the other hand, the expression of Ki-67 in breast cancer cells is similar for cells growing in both complete and FBS-deprived medium (Figure A-B of S1 File, right side), thus in strong agreement with the findings of Fig 1.

### Transcriptional evaluation of Ki-67 expression

**Fig 2:**
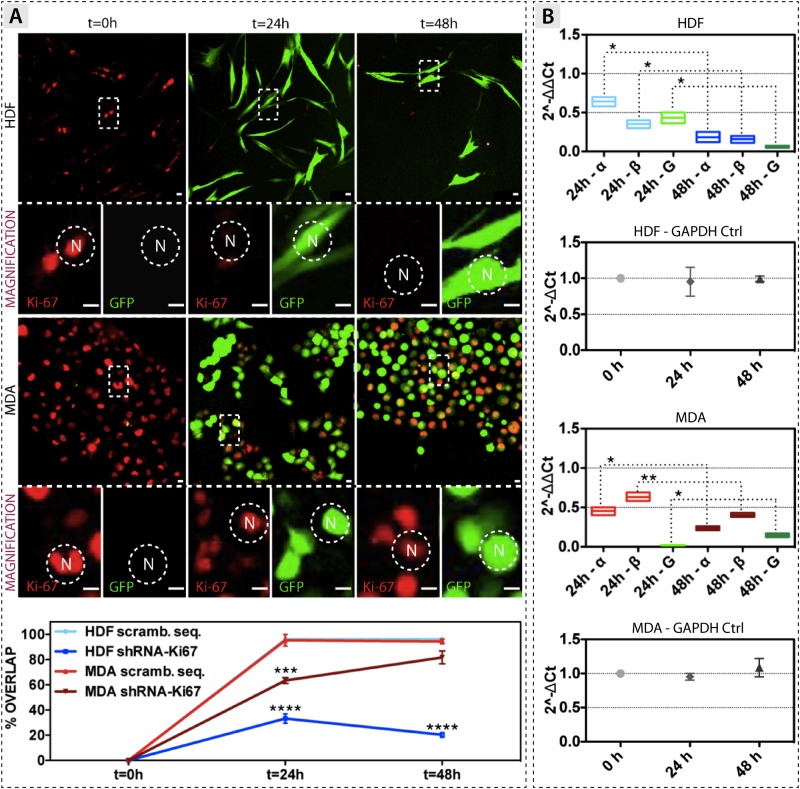
Ki-67 gene knock-down experiment. **(A)** Confocal analysis of Ki-67 expression in HDF and MDA-MB-231 cells after shRNA knock-down. Bottom part: graph showing the Ki-67 protein quantification. **(B)** RT-qPCR quantification of the α and β splice variants of Ki-67, and GAPDH (G), after virus knock-down. The green graph represents the internal control GAPDH. For both cell models the relative quantification of GAPDH internal control (Ctrl) during Ki-67 gene knockdown experiments is also reported. (t-test, p-value * P ≤ 0.05; ** P ≤ 0.01; *** P ≤ 0.001; **** P ≤ 0.0001). Scale bar: 15 μm.

To further explore weather the regulation of Ki-67 expression is controlled at transcriptional level, we carried out a Ki-67 gene knock-down experiment. Cells were transfected with a lentivirus codifying for short hairpin RNA (shRNA) involved in the transcriptional degradation of both the α and β splice variants (Fig 2). HDF cells do not display the shRNA-related GFP reporter gene immediately after viral infection (t = 0 hours), while the expression of Ki-67 is evident as red spots (Fig 2A). After 48 hours, fibroblasts are completely infected, as confirmed by the strong green signal (reporter gene for the shRNA), combined with the down-expression of Ki-67 (the red spots completely lack in this case). These outcomes are surprisingly different in MDA-MB-231 breast cancer cells. The viral infection with shRNA is not enough to down-regulate Ki-67, which is still present after 48 hours of treatment (red signals of Fig 2A). At the same time, the expression of the GFP confirms the good lentivirus-mediated transfection. Signal quantification (images analyses) also confirmed the strong down-expression of Ki-67 in transfected HDF, together with the impossibility to repress the transcript expression in transfected MDA-MB-231 cells (bottom part of Fig 2A). In this latter case, the quantified signals are very close to those belonging to the control cells, which have been infected with a not codifying (scrambled) sequence.

We then analysed the regulation of the two splice variants upon Ki-67 transcript degradation (the shRNA is not able to distinguish between the two splice variants, and it degrades equally both subunits). The Ki-67 transcript is significantly decreased in HDF after 48 hours of viral infection, with a silencing efficiency of *c*.*a*. 88% and 92% for the α and β variants, respectively (RT-qPCR analyses of Fig 2B). The Ki-67 transcript is also significantly decreased after 48 hours of viral infection in MDA-MB-231 cells, where we quantified a silencing efficiency of about 86% and 88% for the α and the β splice variants, respectively (Fig 2B).

The ΔCt quantification (Fig 2B) further corroborates the quality of the analyses. In fact, both HDF and MDA-MB-231 show that the internal control gene glyceraldehyde 3-phosphate dehydrogenase (GAPDH) is not affected during these knockdown experiments. Details of the Ki-67 introns and exons regions, and the two possible splice variants, are shown Figure B of S1 File.

### Ki-67 expression in cancerous and non-cancerous cells before and after nutrients deprivation

**Fig 3:**
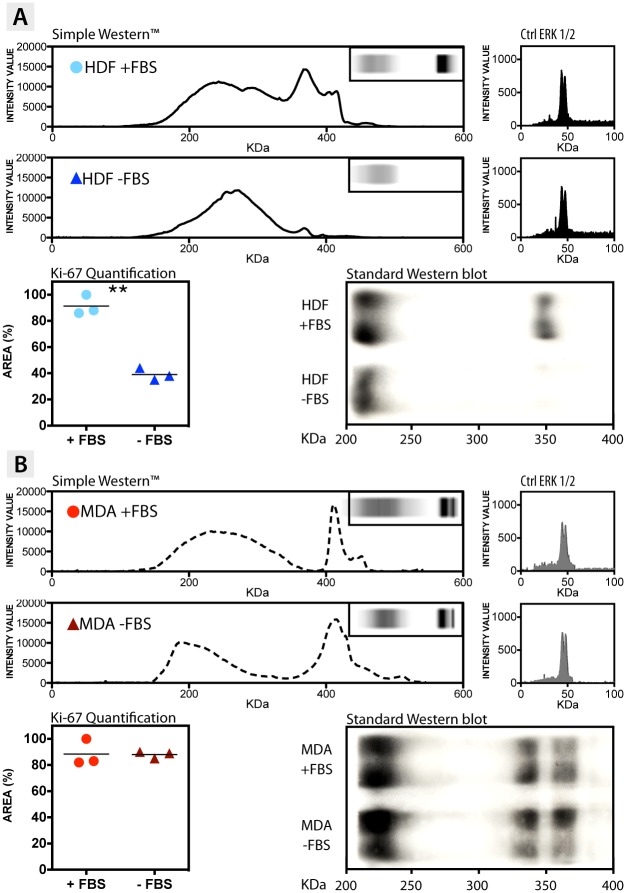
Ki-67 protein quantification. Simple Western^™^, Standard Western, and quantification profile of Ki-67 in **(A)** HDF and **(B)** MDA-MB-231 breast cancer cells, growing in both media supplemented (+FBS) and lacking of FBS (-FBS). In the figure it is also reported the Simple Western^™^ quantification profile of the internal control ERK 1/2 in HDF and MDA-MB-231 breast cancer cells, before and after FBS deprivation. (t-test, p-value * P ≤ 0.05; ** P ≤ 0.01; *** P ≤ 0.001; **** P ≤ 0.0001).

To have a deeper insight into the molecular bases of Ki-67 expression, and to shed light on the different mechanisms of Ki-67 post-transcriptional and post-translational regulation, a Western blot assays have been carried out with the automated Simple Western^™^ (or Simon^™^). The expression of Ki-67 was quantified in normal growth condition (+FBS), and after 36 hours of FBS deprivation (-FBS). We confirmed that Ki-67 is present within fibroblast cells grown in complete medium, although the two splice variants are not noticeable with such method (Fig 3). This is highlighted in both the profile of expression, where the relative peak of the protein is present, and in the protein run lane in the inset (the dark spot above the profilometer analyses). On the other hand, Ki-67 is not expressed in HDF undergoing 36 hours of serum deprivation, as confirmed by the absence of both the relative intensity peak and the protein band (Fig 3A). We quantified that quiescent fibroblasts have *c*.*a*. 60% less amount of protein, compared to the same proliferative cells (graph of Fig 3A). However, this represents a rather underestimation since the software detects also the signal coming from the background region of the lane. Concerning the MDA-MB-231 cells, the expression of Ki-67 is constitutive/constant in the presence of FBS, as well as after 36 hours of serum starvation. This is evident in the Simple Western^™^ quantification profile (Fig 3B), together with the relative protein band spot in the inset, which both confirm the significant presence of Ki-67. Despite the case of HDF, the α and β splice variants were detected in this case. The quantification analysis (Fig 3) also assessed the similar amount of the protein, either when the breast cancer cells are pushed to a quiescent state (-FBS) and when they are in normal conditions (+FBS). To further validate the data obtained from the automated Simple Western^™^, the ERK 1/2 extracellular-signal-regulated kinase was quantified in the same cellular samples. ERK 1/2 are well conserved proteins that are implicated in essential functions for the cell maintenance and, for this reason, they were chosen as internal controls [16]. As shown in Fig 3A and 3B, ERK 1/2 is equally expressed for both models (HDF and MDA-MB-231), for all the conditions tested. To corroborate the Simple Western^™^ analyses, standard Western blot assays were also performed, confirming the results reliability between different methods (bottom-right side of Fig 3A and 3B).

### Ki-67 α and β splice variant expression as a function of the nutrients deprivation

**Fig 4:**
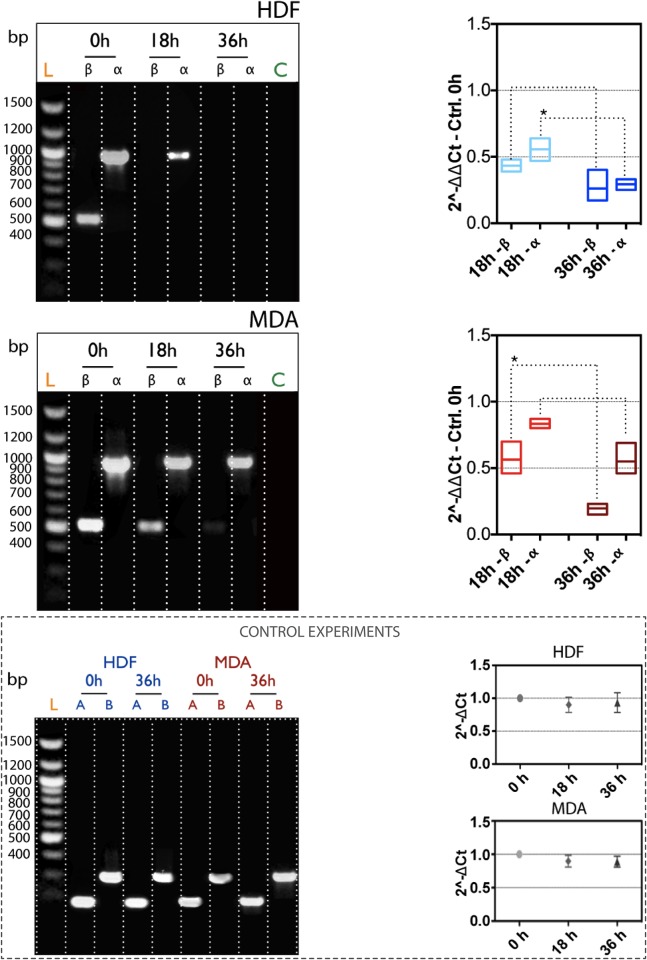
Ki-67 mRNA quantification. RT-PCR (left column), and RT-qPCR (right column) quantification of the α and β splice variants of Ki-67, as a function of nutrient starvation in HDF and MDA-MB-231 cells. Bottom figure: (left) RT-PCR analysis of β-actin (A) and GAPDH (B) as internal controls, before and after nutrient deprivation. (Right) RT-qPCR quantification of the internal control GAPDH after serum starvation. (t-test, p-value * P ≤ 0.05; ** P ≤ 0.01; *** P ≤ 0.001; **** P ≤ 0.0001).

We then explored the relations between the expression of the two splice variants of Ki-67 and the nutrient conditions (presence and absence of FBS). This approach led us to disclose which subunit plays a key role in controlling the cell shifting in the different sub-stages of the interphase. According to our results, both subunits are present in HDF cells at time zero (PCR investigations in Fig 4, top-left side). After 18 hours of serum starvation, the α is still present, although the expression level is significant less comparing to time zero, while the β is completely down regulated. Both the α and β splice variants are then completely unexpressed in HDF cells after 36 hours of nutrient starvation (-FBS). On the other hand, the expression of mRNA for both the α and β subunits of Ki-67 does not undergo significant modifications after 18 hours in MDA-MB-231 (Fig 4A, bottom-left). However, after 36 hours of starvation, only the β subunit seems to be slightly down-regulated as compared to the α, which is significantly expressed. All these PCR outcomes have been further validated by means of quantitative PCR analyses (Fig 4, right side). In particular, the ΔΔCt displays that the mRNA level for the β splice variant (value < 0.5) is less than the transcript of the α (value > 0.5), for HDF cells undergoing 18 hours of incubation with growth medium lacking of serum. Upon 36 hours of starvation, the ΔΔCt value for both the α and β variants is significant low, confirming that control fibroblasts are able to regulated the down-expression of Ki-67 when they lie in the stationary phase of the interphase. This condition is significant different in the breast cancer cells, where the FBS deprivation does not lead to a down-regulation of Ki-67. MDA-MB-231 cells possess higher level of α and β variants with respect to the HDF (0.4<ΔΔCt<0.7 for the β, and 0.6<ΔΔCt<0.9 for the α, respectively) after 18 hours of deprivation. Upon longer starvation time (36 hours), the mRNA level for the β splice variant is significantly down-regulated, while the transcript for the α variant is almost unvaried. However, the most interesting data here is that both the PCR and qPCR confirm a different regulation of the two splice variants, as a function of nutrient conditions (and proliferation indeed). In particular, the α seems to be the molecular switch for regulating the proliferation. A further validation comes from the ΔCt quantification, an important parameter concerning the internal reference control gene GAPDH. Fig 4 reports that there is not a significant difference in the level of ΔCt signal in fibroblasts and breast cancer cells during starvation time.

### Molecular mechanisms of Ki-67 post-translational regulation

**Fig 5:**
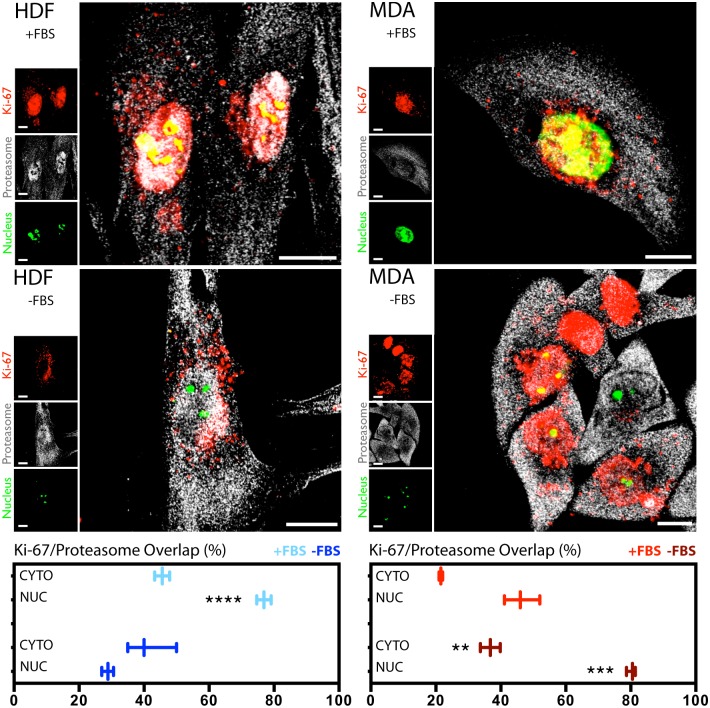
Ki-67 and proteasome interaction. Confocal assays and quantification graphs (on the bottom of the picture) of the distribution and interaction between Ki-67 and the proteasome system, in both HDF (left column) and MDA-MB-231 cells (right column). The analyses were carried out with and without the presence of FBS. (t-test, p-value * P ≤ 0.05; ** P ≤ 0.01; *** P ≤ 0.001; **** P ≤ 0.0001).

In addition to the control over the transcriptional expression of Ki-67, we explored the possibility of post-translational regulations of the protein. To do this, we carried out confocal imaging investigations to understand the interaction between Ki-67 and the proteasome system, which is one of the most important cellular quality control mechanisms of proteins [17, 18]. We explored this proteasome regulation both at the nuclear and cytosolic level. HDF cells grown in complete medium display most of Ki-67 and the proteasome system co-localising within the nucleus (Fig 5). A quantification of the co-localisation event (bottom part of Fig 5) confirms that Ki-67 interacting with the cytosolic proteasome is about half of the same protein interacting with nuclear proteasome (normalised fluorescence signal). Hence, the Ki-67-proteasome interaction resulted much stronger at nuclear level comparing to the same interaction occurring within the cytosol. On the other side, there is not a significant difference in the co-localisation signal between Ki-67 and the proteasome system both at the cell nucleus and the cytosol, for fibroblasts undergoing 18 hours of serum starvation (-FBS). However, serum deprivation leads to an overall lower level of signal from Ki-67 in the nucleus, indicating that most of the protein has already been degraded. MDA-MB-231 cells display high levels of Ki-67 in both the cytosol and nucleus (although here it resulted more abundant), both for starved and non-starved cells (Fig 5). The quantification of co-localisation events (bottom-right part of Fig 5) confirms that the Ki-67-proteasome interaction is much stronger at nuclear level where the Ki-67 is mostly present, comparing to the same interaction in the cytosol. This has been found to be independent on the both conditions tested, as the overlap value is ~40% lower in the cytoplasm as compared to the overlap value of the nucleus. This would indicate probably that cancer cells fail in the correct Ki-67 degradation.

**Fig 6:**
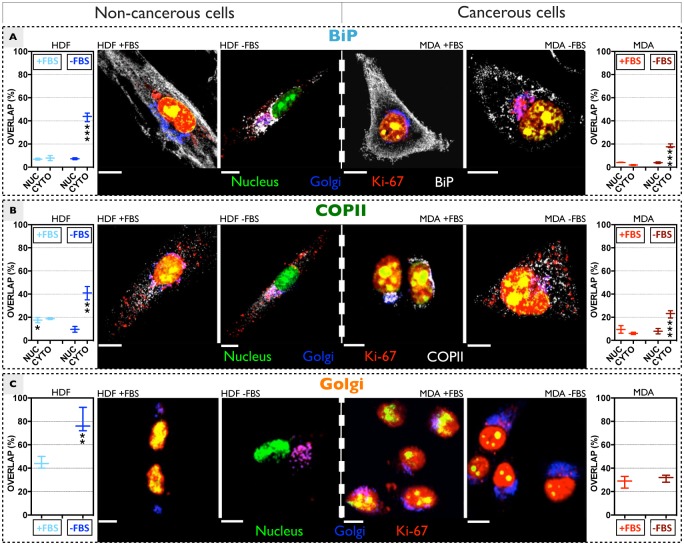
Ki-67 extranuclear distribution. Confocal assays of the distribution and interaction between Ki-67 and BiP **(A)**, COPII **(B)** and Golgi **(C)**, in HDF (left column) and MDA-MB-231 cells (right column), before and after serum deprivation. The quantification graphs (on the left and right sides of the picture) show the overlap signal between Ki-67 and the complex of interest, for HDF and MDA-MB-231 growing in both media supplemented and lacking of FBS. For a single channel analysis, see Figures D-G of S1 File. (t-test, p-value * P ≤ 0.05; ** P ≤ 0.01; *** P ≤ 0.001; **** P ≤ 0.0001).

We have also important evidences of a second possible regulation pathway of Ki-67, which is much more related to the cell secretory system. In particular, we observed the presence of the protein at the ER and Golgi level, a quite unexpected outcome. To this respect, we first explored a possible co-localisation between Ki-67 and the ER molecular chaperon Binding immunoglobulin protein (BiP). This chaperon is specifically located within the lumen of the ER, and it is responsible for binding the nascent polypeptides which are emerging in the lumen of the ER during co-translational translocation [19–21]. We firstly analysed the co-distribution of Ki-67 and BiP in active fibroblasts (Fig 6A, left side). We quantified only a small portion of the protein co-localising with BiP, whose fluorescence signal is randomly spread throughout the cytosol (Fig 6A). The interaction between Ki-67 and BiP becomes higher in 18 hours starved HDF (Fig 6A). On the other hand, a significant difference in the Ki-67 and BiP coupling in starved and non-starved breast cancer cells is less evident. We observed, in fact, a poor co-localisation between the two proteins (Fig 6A, right side). In addition, BiP is randomly distributed in both the conditions tested, showing low accumulation at the Golgi compartment in FBS deprived cancerous cells. It is worth also mentioning that, after 36 hours of starvation time, all the extranuclear Ki-67 is in the Golgi apparatus, where it may undergo further modifications/regulations. This co-localisation assay would support the hypothesis that Ki-67 could be translocated within the ER. We thus carried out another experiment aiming to understand if the presence of the protein in the ER is due to a cytosol-to-ER translocation of newly synthesised Ki-67. Nascent polypeptides are translocated into the ER lumen space via the translocator Sec61. Hence, a resulting co-localisation between the two proteins would indicate a mechanism addressed to the import of nascent Ki-67. However, no relevant interactions are detected between Sec61 and Ki-67 (Figure C of S1 File). This excludes the hypothesis that Ki-67 is imported into the ER via Sec61, and indicates that the presence within the ER is related to Sec61-independent translocation (not addressed in this work).

We then investigated the possible interaction of Ki-67 with the cytoplasmic coat protein complex II (COPII), mainly because of a preliminary observation of a fully Ki-67 loaded Golgi apparatus, after a long HDF starvation time (Fig 6A). COPII is the main constituent of specific vesicles, which bud from the endoplasmic reticulum and transport their cargo (secretary proteins) to the Golgi system [22–24]. Quantification of fluorescence overlap suggests that Ki-67 and COPII are interacting in both quiescent and proliferating fibroblasts (Fig 6B, left). Concerning MDA-MB-231 cells seeded with a complete media, the fluorescence signal of COPII vesicles displays a very well defined distribution pattern around the nucleus, together with a poor interaction with Ki-67 (Fig 6B, right side). Upon 18 hours serum starvation, COPII is widely spread within the cytosol instead, and the overlap signal with Ki-67 is higher as compared to non-starved cells.

We finally investigated the Ki-67 delivery into the Golgi. Despite the protein was detected in the Golgi system in both quiescent and active fibroblasts, its accumulation seems to be higher upon FBS deprivations (Fig 6C, left side). On the other side, nutrient conditions do not influence the distribution and presence of Ki-67 in the Golgi of MDA-MB-231 cells (Fig 6C).

In addition, we investigated two other possible cellular clearance/degradation mechanisms for Ki-67: the lysosome and the autophagosome (Figure D of S1 File). However, we did not found any interaction between the protein and these two degradation machineries.

## Discussion

The possibility to exploit Ki-67 as effective biomarker for cancer prognostic is constantly debated by the medical community and, after more than two decades, contrasting opinions are still evident. This may be ascribed to an overall lack of systematic studies on the topic, which mainly focused on the intranuclear pathways of Ki-67.

We report two key differences between cancerous and non-cancerous cells: (i) we demonstrated that cancerous and non-cancerous cells differently control the expression of Ki-67 and its isoforms, (ii) while we did not see any differences in the protein degradation via the proteasome, we noticed considerable differences between the two cells in term protein regulation at the ER/Golgi level. Our data clearly confirm that non-cancerous cells are able to control the down-regulation of Ki-67 during the G_0_/G_1_ phase of the interphase, while cancer cells over-express the protein during nutrients starvation (Figs 1 and 3). Moreover, the protein is still present in cancerous cells even after shRNA silencing of the gene (Fig 2), probably because Ki-67 is more stable herein or, alternatively, its post-translational degradation fails to occur in cancer cells.

We also found a correlation between the progression through the cell cycle and the specific Ki-67 splice variants. In particular, quiescent fibroblasts down-express the β variants only, while both isoforms are always present in cancer cells (Fig 4). This can shed light on some basic mechanisms of cells proliferation, an important topic in cancer research. Hence, these findings strongly support the hypothesis that the design of next generation of anticancer drugs, based on targeting the Ki-67 expression and/or regulation, should consider the possibility to selectively target the α variant only. In fact, this Ki-67 isoform has been defined as the constitutively expressed in cancerous cells, even when these are pushed into a quiescent phase (Fig 4).

Another interesting outcome of our work is the proteasome-related post-translational regulation, occurring both at nuclear and cytosolic level (with different efficiency between the two compartments).

Although it is already known that Ki-67 contains different ubiquitination sites, we show different proteasome degradation pathways between cancerous and non-cancerous cells. In particular, in non-cancerous cells proliferation ending corresponds to effective proteasome degradation of the already present Ki-67. This phenomenon is rather uncontrolled in cancerous cells. Together with the transcriptional control over the mRNA of each splice variant, we believe that the proteasome degradation represents the principal way the cells control the progression within the interphase.

We have also evidences of a possible concomitant “feedback elimination mechanism” of Ki-67, based on the ER/Golgi secretory pathway. We observed an interaction between Ki-67 and the ER luminal protein BiP. Since BiP is demonstrated to be part of the unfolded protein response (UPR) activation clusters [25–28], we assume that Ki-67 can be degraded through this pathway (ongoing work is underway to evaluate this hypothesis). Such presence in the ER is further confirmed by a co-localisation with COPII vesicles and a considerably stronger co-localisation with the Golgi, suggesting a secretion mechanism of the nascent protein. Most notably, the same cannot be said for the breast cancer cells, where, although some signal of Ki-67 is still visible in the Golgi, the overall levels are not comparable to the non-cancerous cells. The presence of the protein in the Golgi has important implications especially in oncology. Alteration in Golgi-mediated post-translational modification, such as glycosylation, can regulate cancer progression. This, together with the conventional proteasome degradation, suggests that Ki-67 is very carefully regulated and its homeostasis is quite different between the two cells models.

**Fig 7:**
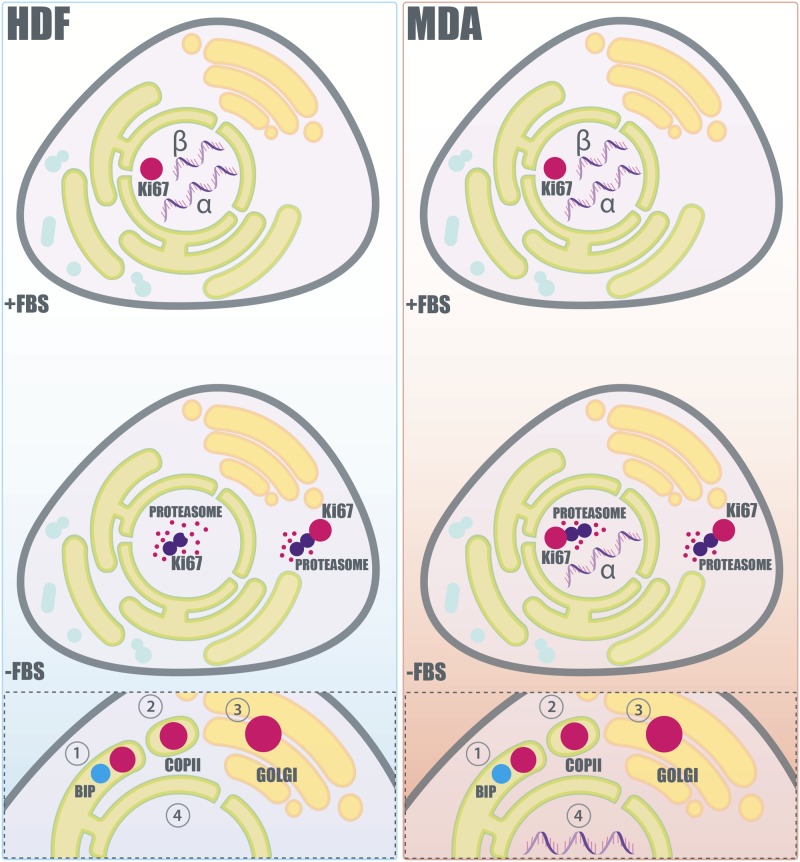
Proposed molecular regulation and extranuclear pathway of Ki-67. Scheme representing the proposed Ki-67 regulation in non-cancerous (HDF) and cancerous cells (MDA-MB-231) before and after serum deprivation. In non-cancerous cells (left column), prolonged serum deprivation produces the down-expression of both the α and β splice variants of Ki-67. Furthermore, the already expressed Ki-67 is degraded *via* the proteasome. On the other hand, cancerous cells (right column), subjected at the same starving conditions, show the continuous expression of the α splice variant of the protein. Moreover, the level of the detected Ki-67 in cancerous cells is not affected overtime by the degradative action of the proteasome. The “feedback elimination mechanism” of Ki-67 degradation, which is related to the ER-Golgi secretory machinery, is schematised in the box at the bottom of the figure. In this proposed mechanism, Ki-67 is initially transferred to the ER, where it colocalises with BiP **(1)**. Subsequently, Ki-67 buds from the ER into specific COPII coated vesicles **(2)**, which transport their cargo to the Golgi apparatus **(3)** where Ki-67 may be further recycled and/or degraded. Non-cancer and cancer cells translocate Ki-67 with the same mechanism. However the obtained data indicate that cancerous cells could be characterised by a defective ER-Golgi secretory machinery. **(4)** Moreover, the Ki-67 translocation in cancerous cells could be unbalanced by the non down-regulation of the α variant of the protein.

All these findings have been summarised in the scheme in Fig 7, which shows a proposed molecular mechanisms involved in the extranuclear regulation of Ki-67. In particular, proliferating cells possess a high amount of the protein in the nucleus. However, the stasis of non-cancerous cells in the G_0_ of the interphase is due to an effective (i) down expression of both the α and β splice variants, to a (ii) nuclear and cytosolic proteasome degradation, and possibly to (iii) the synergic activation of the feedback elimination mechanisms where Ki-67 is delivered to the ER first, and the Golgi later. Cancerous cells degrade the protein with similar mechanisms, however the α variant is not down-regulated, and both the primary and feedback mechanisms could be defective. Hence, the fine balance between expressed and degraded Ki-67 results hindered, and cancer cells are not able to control the transfer between a quiescent-to-proliferative state of the interphase.

Taken together, all these data demonstrates the importance of Ki-67 in the replication activity of cells. We believe that this topic should be significantly revisited especially in the viewpoint of the isoform-dependent proliferation and of its extranuclear regulation. The next efforts should be then focused on a pragmatic screening of any possible intracellular biomolecular target that may control the expression/degradation of a specific Ki-67 splice variant.

## Materials and methods

### Cell culture

Primary human dermal fibroblasts (HDF), human breast cancer cell line MDA-MB-231 (MDA), human cervix adenocarcinoma cells (HeLa), and hypopharyngeal carcinoma cells (FaDu) cells were purchased from ATCC^®^. HDF, HeLa, and FaDu cells were cultured and maintained using Dulbecco's Modified Eagle Medium (DMEM) (Sigma-Aldrich^®^) containing: 10 (v/v) fetal calf serum, 2 mM L-glutamine, 100 mg/ml streptomycin and 100 IU/ml penicillin (Sigma-Aldrich^®^). Cells were cultured at 37°C/95% air/5% CO_2_. Human umbilical vein endothelial cells (HUVECs) were purchased from Life Technologies^™^ and cultured using Medium 200 with Low Serum Growth Supplement (LSGS) (Life Technologies^™^, USA) and maintained as reported for the previous cells. MDA-MB-231 cells were cultured in RPMI 1640 medium (Lonza^®^), containing: 10 (v/v) fetal calf serum, 2 mM L-glutamine, 100 mg/ml streptomycin and 100 IU/ml penicillin (Sigma-Aldrich^®^). Cells were periodically sub-cultured using Trypsin-EDTA solution 0.25% (Sigma-Aldrich^®^) for the detachment process and centrifuged at 2000 rpm for 5 min for the pellet collection.

The cellular synchronisation was obtained by incubating the cells with serum-deprived medium, at different time points (18, 24, 36 and 48 hours), depending on the experimental conditions.

### Confocal and imaging analysis

For the evaluation of distribution and interactions between different proteins, cells were initially seeded in glass bottom dishes (35 mm diameter-IBIDI^®^) at a density of 8 x 10^3^ cells per well, grown for 24 hours in complete medium, and finally analysed by means of confocal microscopy.

The protein-protein interactions were investigated under confocal microscope (Leica TCS SP8), and the cellular samples were processed with a standard immunofluorescence protocol: fixation with formaldehyde 3.7% for 10 min, permeation with 0.1% Triton X-100 in phosphate buffered saline (PBS) for 10 min, block with bovine serum albumin (BSA) 2% for 2 hours.

Ki67 protein was directly investigated through a mouse IgG anti-Ki-67, conjugated with Brilliant Violet 421^™^ dye (350505; BioLegend^®^ - λ_ex_ = 405 nm, and λ_em_ = 421 nm). The Golgi compartment staining was achieved by treating cells with a modified baculovirus expressing a fusion construct of a Golgi marker and a red fluorescent protein (CellLight^®^ Golgi-RFP—C10593; Life Technologies^™^- λ_ex_ = 540 nm, and a λ_em_ = 625 nm). All the other confocal characterisations were carried out using specific unlabelled primary rabbit IgG, incubating the latter with the cellular sample for 2 hours. Proteins were then enlighten by secondary DyLight^™^ 649 donkey anti-rabbit IgG (406406; BioLegend^®^ - λ_ex_ = 655, nm and a λ_em_ = 670 nm) which was incubated within the sample overnight. The proteasome detections was achieved with a specific IgG recognising the 20s proteins subunit (ab3325; Abcam^®^). The COPII-vesicles detection was performed using an IgG recognising the Sec31A subunit (HPA005457; Sigma-Aldrich^®^). The BiP investigation was obtained through an antibody recognising the specific N-term AA sequence of the protein (G9043; Sigma-Aldrich^®^). Finally, the nucleic acid staining was obtained with the molecular probe SYTO^®^9 (Life Technologies^™^ - λ_ex_ = 490 nm and a λ_em_ = 525 nm). The average Ki-67 overlap value intensity with SYTO^®^9 and the investigated proteins was calculated with an *ad hoc* developed MATLAB^®^ script.

### Western blot analysis

For the western blot analysis, the cells were cultured in T75 flasks and lysed with radio-immunoprecipitation assay (RIPA) buffer (20 mmol/L Tris, pH 7.5, 150 mmol/L NaCl, 1% Nonidet P-40, 0.5% sodium deoxycholate, 1 mmol/L EDTA, 0.1% SDS) (Sigma-Aldrich^®^), containing complete mini protease inhibitors (Roche^®^).

The total protein initial concentration was calculated using the bicinchoninic acid assay (BCA) [29] (Thermo Fisher Scientific) and thus normalised for all the cellular samples. All the samples were denatured for 5 min at 95°C prior the western size-based assay or the standard western blot assay.

### A. Western size-based assay

The western blot analysis for the KI-67 protein quantifications was performed with an automated western size-based assay (ProteinSimple-Simon^™^), following the company standard protocol and using 20 μg of protein per sample. The specific antibody applied against Ki-67 was purchased from Abcam^®^ (ab15580), while the ERK 1/2 positive control antibody was provided by the Simon^™^ analysis kit. This automated Western blot technology is based on capillary-electrophoresis-SDS (CE-SDS). The protein identification is performed upon incubation with a primary antibody, followed by an immunodetection based on a horseradish peroxidase (HRP), which is conjugated to the secondary antibody, together with a chemiluminescent substrate for the binding detection. The automated Simple Western^™^ combines several advantages, including the possibility of actual quantification and the higher reproducibility of results over time and between different users [30, 31]. The analysis was performed in both the cell model tested.

### B. Standard western blot assay

NuPAGE4-12% TrisGels (Life Technologies^™^) was used to achieve the protein fractions separation. Each sample (20 μg of protein in a volume of 40 μl) was loaded into the gel well and their alignment on the gel front was achieved applying a voltage of 100 V for 15 min. Subsequently, the protein fractions separation was obtained with a voltage of 180 V per 150 min.

After this step, the gel trapped proteins were transferred thanks to the iBlot instrument (Life Technologies^™^) to a nitrocellulose membrane. The transfer was performed applying a voltage of 100 V per 60 min.

Subsequently, the membrane was blocked with 1 hour incubation at room temperature with 5% (w/v) milk powder in Tris-buffer with 0.5% (v/v) Tween 20 (blocking solution). The membrane was stained for Ki-67 by overnight incubation at 40°C with anti-Ki-67 antibodies diluted in blocking solution (ab 15580; 1:500; Abcam^®^, UK). The membrane was then washed three times with 1X PBS and incubated with horse-radish peroxidase (HRP) secondary antibodies, also diluted in blocking solution (1:10000; Cell Signaling Technologies). Finally, the membrane was treated for 5 min with the enhanced chemiluminescence kit (Thermo Fisher Scientific). The photographic development of the obtained results was performed in a dark room exposing a photographic film to the obtained membrane for 1 min. Afterwards, the photographic film was immersed for 5 seconds in a developing solution and fixed by placing it for 30 seconds in a fixing solution.

### Fluorescence cytometry (FC) analysis

This technique was used for the cell cycle analysis and for the quantifications of Ki-67 expression. The cell cycle analysis was performed by applying a cellular staining protocol with propidium iodide (PI) [32]. The Ki67 quantification was obtained by using a classic cellular fixation and permeabilisation protocol with 70% ethanol. A mouse IgG anti-Ki67, conjugated with Alexa Fluor^®^ 647 dye (652408; BioLegend^®^ - λ_ex_ = 635 nm, and a λ_em_ = 670 nm), was applied for the FC analysis.

### Reverse transcription polymerase chain reaction (RT-PCR), and PCR assays

Cultured cells were lysed and prepared as described for the western blot samples preparation. Total RNA was collected by using RNeasy Mini Kit (Qiagen). RNA concentration was measured with NanoDrop spectrophotometer. Complementary DNA (cDNA) was synthesised from every 1 μg of total mRNA in 20 μL volume per tube with QuantiTect Reverse Transcription Kit (Qiagen). The samples were then run in a standard agarose gel (1%). For the PCR analyses, GAPDH was used as a reference gene, and the two Ki67 isoforms (α and β) were analysed using the following list of primers:

GAPDH:

forward 5’-CAGCCTCAAGATCATCAGCA-3’,

reverse, 5’-GTCTTCTGGGTGGCAGTGAT-3’;

Ki67 long isoform (α):

forward 5’-GAAAGCTCAAGATTCCAAGGC-3’,

reverse 5’-GCCCAATTTCTCAGGCTTGC-3’;

Ki67 short isoform β:

forward 5’-TATCAAAAGGAGCGGGGTCG-3’,

reverse 5’-TTGGGGCTTCTCCCCTTTTG-3’.

### Real time-quantitative polymerase chain reaction (RT-qPCR)

Cell lyses and cDNA preparation was performed as describe for the RT-PCR analysis. Also in this case, two Ki-67 isoforms (α and β) were examined, using GAPDH as an internal control gene.

The following lists the primers sequence used for the RT-qPCR:

GAPDH:

forward 5’-ACAGTCAGCCGCATCTTCTT-3’,

reverse, 5’-ACGACCAAATCCGTTGACTC-3’;

Ki67 long isoform (α:

forward 5’-TGTTGGTCTCGCGTAAGTCAA -3’,

reverse 5’-CAGACTCCACGTCTCTTCCC-3’;

Ki67 short isoform (β:

forward 5’-AGCACGTCGTGTCTCAAGAT-3’,

reverse 5’-GGTATTCCCTCACTCTCATCAGG-3’.

Quantitative analysis was assessed with QuantiTect SYBR Green RT-qPCR Kit (Qiagen). The amplification process was done in 20 μL/tube, using the following steps: 95°C for 5 min to make active the DNA Polymerase, followed by 40 cycles of 95°C (10 seconds) for denaturation, and 60°C (30 seconds) for combined annealing and extension for all primers. Melting curve was also acquired, to analyse the sample quality, from 55°C to 99°C, by increasing of 1°C/min. Data were analysed via ΔΔCt value. 2^-ΔΔCt^ was calculated as follows: ΔCt = Ct_Ki67_-Ct_GAPDH_; ΔΔCt = ΔCt_(treated)_-ΔCt_(control)_.

### shRNA genes knockdown

The genes knockdown analysis was performed using the Thermo Scientific^™^ GIPZ lentiviral^™^ system, following the company protocol. Briefly, HDF and MDA-MB-231 cells were treated with shRNA loaded viruses, and cultured in serum-free medium respectively with 12 x 10^6^ transducing units per ml (TU/ml) and 3.6 x 10^6^ TU/ml for 6 hours. Subsequently, the medium was substituted with fresh one and the cells were incubated for 0, 24 and 48 hours at 37°C/95% air/5% CO_2_. The effectiveness of the shRNA gene translation was evaluated under confocal microscopy (Leica TCS SP8) detecting the expression of the TurboGFP reporter gene (λ_ex_ = 490 nm, and a λ_em_ = 525 nm), which is also present on the pGIPZ lentiviral vector. Afterward, the mRNA gene target expression was quantified with (A) Confocal microscopy analysis and (B) RT-qPCR. As negative control, a non-silencing virus, expressing the scrambled sequence TCTCGCTTGGGCGAGAGTAAG was used; Ki-67 gene was targeted with the following shRNA antisense sequence: TCCTTAGGAGTCTGTAGCT (clone Id: V3LHS_387958). Finally, the antisense sequence CCTCATTTCCTGGTATGACAA was used to silence the positive control GAPDH.

### Statistical analysis

The statistical comparison between two group of data obtained in experiments such as FC characterisation, Simple Western^™^ quantification, RT-qPCRs, confocal and colocalisation experiments was performed using a t-test. The statistical comparison between more than two groups of data obtained in experiments such as the confocal quantitative analysis (Fig 1) was performed using a two-way ANOVA test. For both statistical comparisons (t-test and two-way ANOVA), the significance level was set-up with p<0.05 and the calculations were performed using GraphPad Prism 6. The experimental error is expressed as standard deviation (N = 3).

## Supporting information

S1 File **Fig A. Fluorescence Cytometry (FC) analyses. A)** Analyses of the cell cycles before and after serum deprivation for HDF (left) and MDA (right) cells. **B)** KI-67 expression as a function of the different stage of the cell cycle. **Fig B. The MKI67 gene.** Schematic representation of the MKI67 gene (including the intronic and exonic regions), and of the two splice variants. **Fig C. Sec61 co-localisation analyses.** Confocal analyses of the distribution of Ki-67 and Sec61 in HDF and MDA-MB-231 cells, growth in complete or serum deprived medium. **Fig D. Ki-67 co-localising with lysosomes and authophagosomes.** Confocal co-localisation analyses to explore the possible lysosomial and autophagosomal degradation of Ki-67. **Fig E. Ki-67 and the Golgi.** Confocal co-localisation analyses of interaction between Ki-67 and the Golgi complex, the proteasome system, and the nucleus. **Fig F. Confocal analyses showing the co-localisation between BiP and KI-67.** Here we show all the different channels split each other from Fig 6 in the main article for a better visualisation. **Fig G. Confocal analyses showing the co-localisation between COPII and KI-67.** Here we show all the different channels split each other from Fig 6 in the main article for a better visualisation. **Fig H. Confocal analyses showing the co-localisation between the Golgi apparatus and KI-67.** Here we show all the different channels split each other from Fig 6 in the main article for a better visualisation.
(DOCX)
