# Supplementary material for: The role of the two splice variants and extranuclear pathway on Ki-67 regulation in non-cancer and cancer cells
Source: PLoS One. 2017 Feb 10;12(2):e0171815. doi: 10.1371/journal.pone.0171815 (PMC5302784; doi:10.1371/journal.pone.0171815)
Supplement: S1 File — Fig A. Fluorescence Cytometry (FC) analyses. A) Analyses of the cell cycles before and after serum deprivation for HDF (left) and MDA (right) cells. B) KI-67 expression as a function of the different stage of the cell cycle. Fig B. The MKI67 gene. Schematic representation of the MKI67 gene (including the intronic and exonic regions), and of the two splice variants. Fig C. Sec61 co-localisation analyses. Confocal analyses of the distribution of Ki-67 and Sec61 in HDF and MDA-MB-231 cells, growth in complete or serum deprived medium. Fig D. Ki-67 co-localising with lysosomes and authophagosomes. Confocal co-localisation analyses to explore the possible lysosomial and autophagosomal degradation of Ki-67. Fig E. Ki-67 and the Golgi. Confocal co-localisation analyses of interaction between Ki-67 and the Golgi complex, the proteasome system, and the nucleus. Fig F. Confocal analyses showing the co-localisation between BiP and KI-67. Here we show all the different channels split each other from Fig 6 in the main article for a better visualisation. Fig G. Confocal analyses showing the co-localisation between COPII and KI-67. Here we show all the different channels split each other from Fig 6 in the main article for a better visualisation. Fig H. Confocal analyses showing the co-localisation between the Golgi apparatus and KI-67. Here we show all the different channels split each other from Fig 6 in the main article for a better visualisation. (DOCX) [file pone.0171815.s001.docx]

The role of the two splice variants and extranuclear pathway on Ki-67 regulation in non-cancer and cancer cells

SUPPLEMENTARY MATERIAL

Luca Chierico^1^,* Loris Rizzello^1^,* Lijuan Guan^1^, Adrian S. Joseph^1^, Andrew L. Lewis^2^ and Giuseppe Battaglia^1^

^1^Department of Chemistry, University College London, London, United Kingdom.

^2^Biocompatibles UK Ltd., Farnham Business Park, Weydon Lane, Farnham, United Kingdom.

*: These authors equally contributed to the work

Corresponding authors:

Dr. Loris Rizzello, Department of Chemistry – UCL

20 Gordon Street, WC1H 0AJ London (UK)

e-mail: l.rizzello@ucl.ac.uk

Prof. Giuseppe Battaglia, Department of Chemistry – UCL

20 Gordon Street, WC1H 0AJ London (UK)

e-mail: g.battaglia@ucl.ac.uk


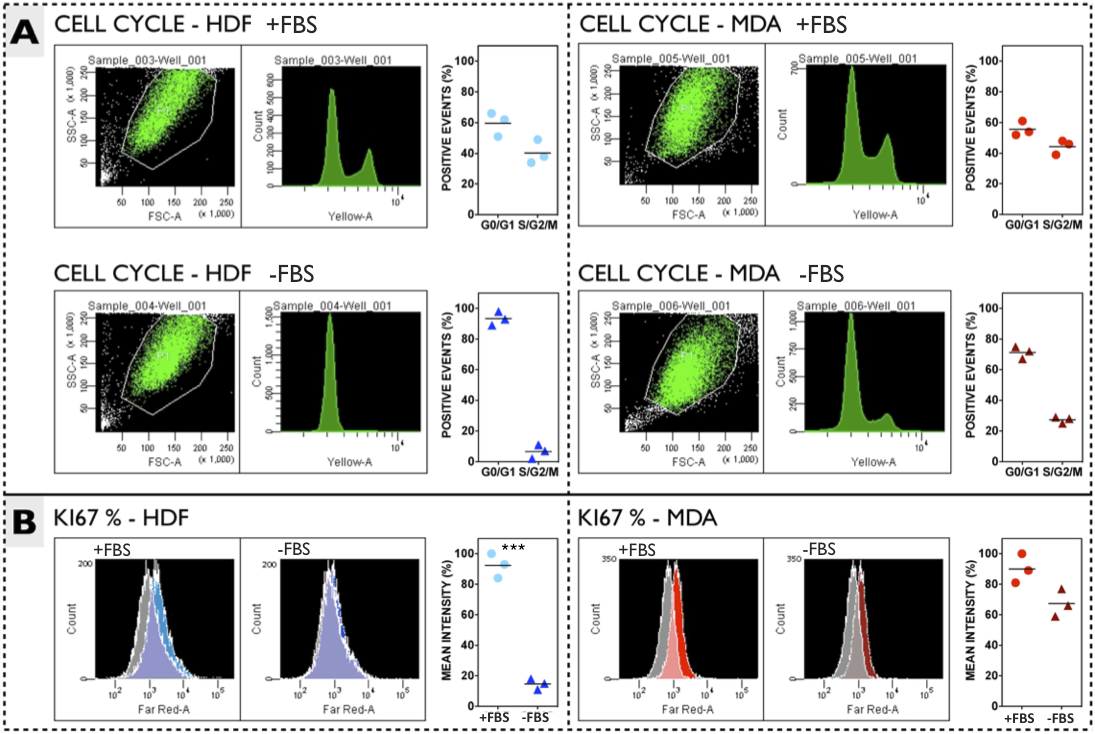
 **Fig A. Fluorescence Cytometry (FC) analyses.** **A)** Analyses of the cell cycles before and after serum deprivation for HDF (left) and MDA (right) cells. **B)** KI-67 expression as a function of the different stage of the cell cycle.

**Fig B. MKI67 gene and splice variants.** Organisation of exons and introns in *MKI67* gene (top), and the splice variant that leads to the formation of the two isoforms (bottom). The difference between the two isoforms is the presence (or not) of the exon 7.


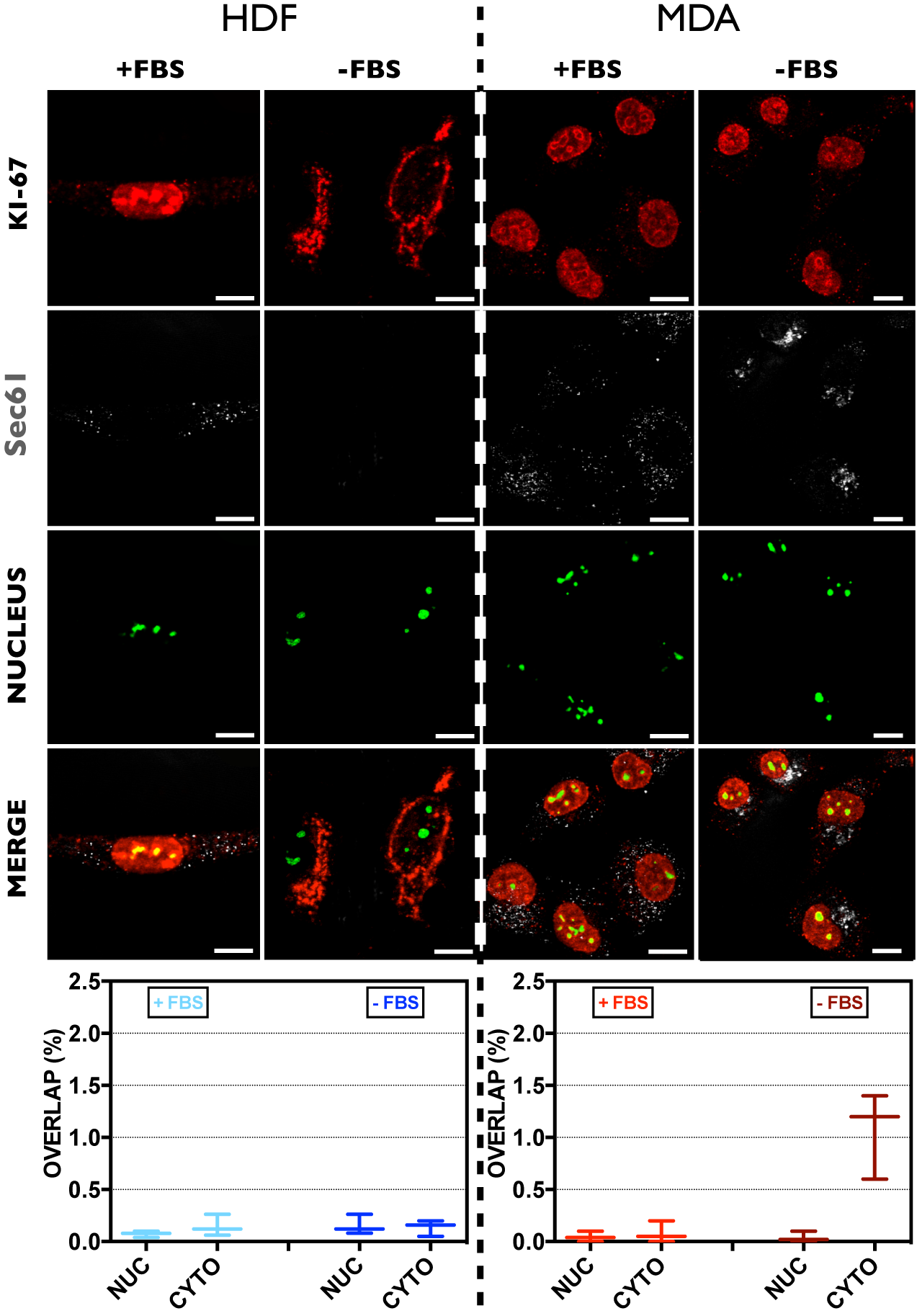


**Fig C. Sec61 co-localisation analyses.** Confocal analyses of the distribution of Ki-67 and Sec61 in HDF and MDA-MB-231 cells, growth in complete or serum deprived medium.


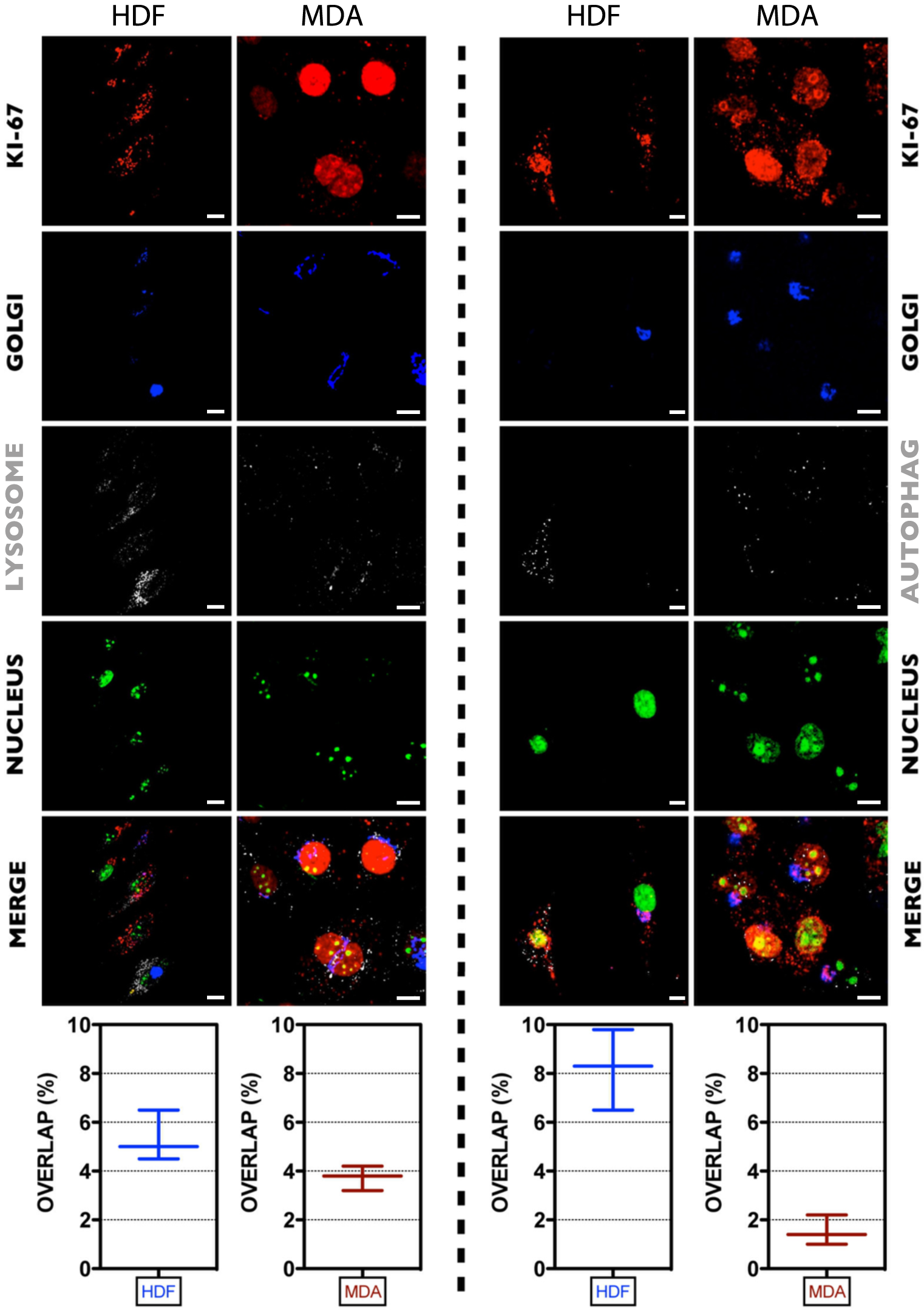


**Fig D. D. Ki-67 co-localising with lysosomes.** Confocal co-localisation analyses to explore the possible lysosomial and autophagosomal degradation of Ki-67.


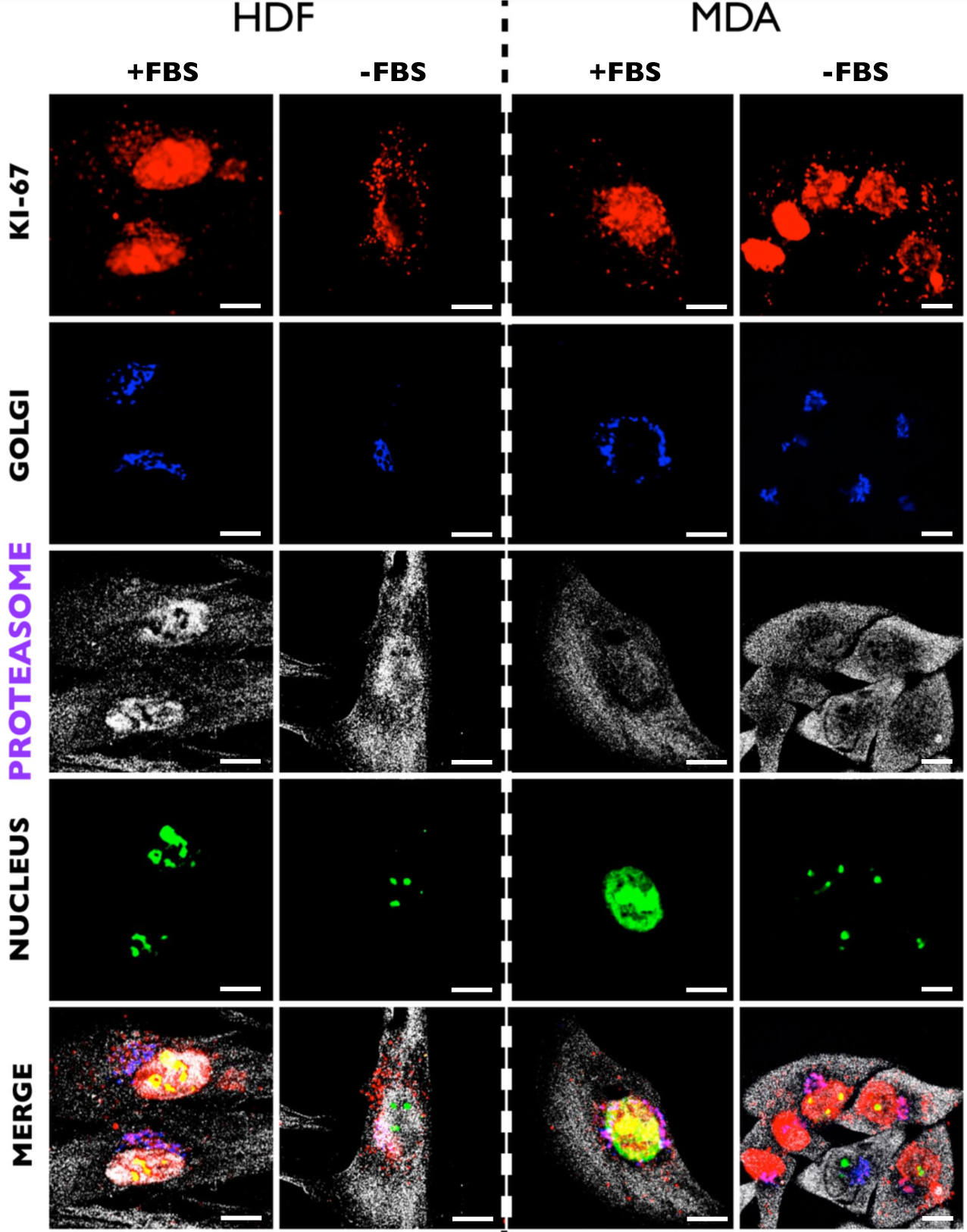


**Fig E. Ki-67 and the Golgi.** Confocal co-localisation analyses of interaction between Ki-67 and the Golgi complex, the proteasome system, and the nucleus


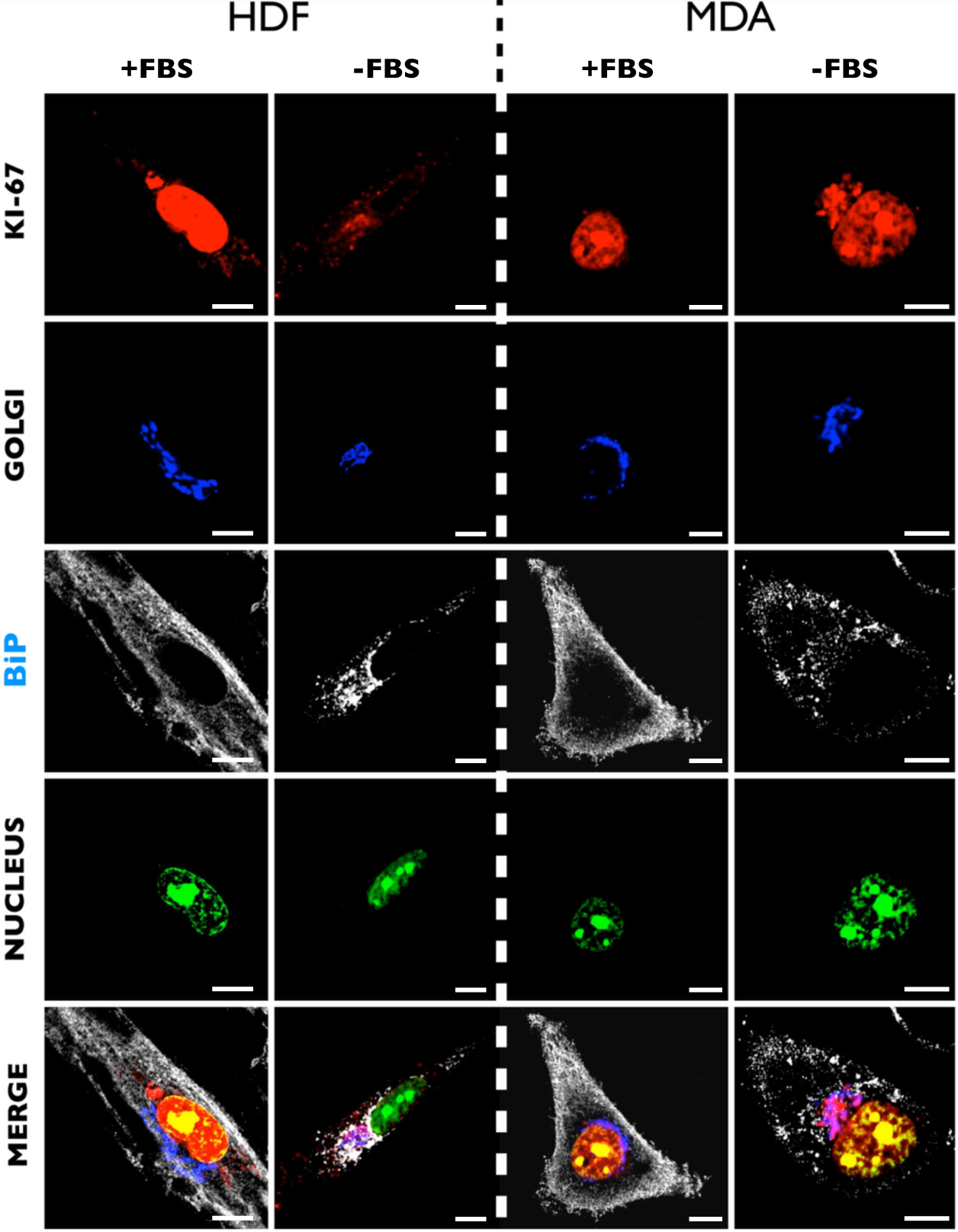


**Fig F. Confocal analyses showing the co-localisation between BiP and KI-67.** Here we show all the different channels split each other from figure 6 in the main article for a better visualisation.


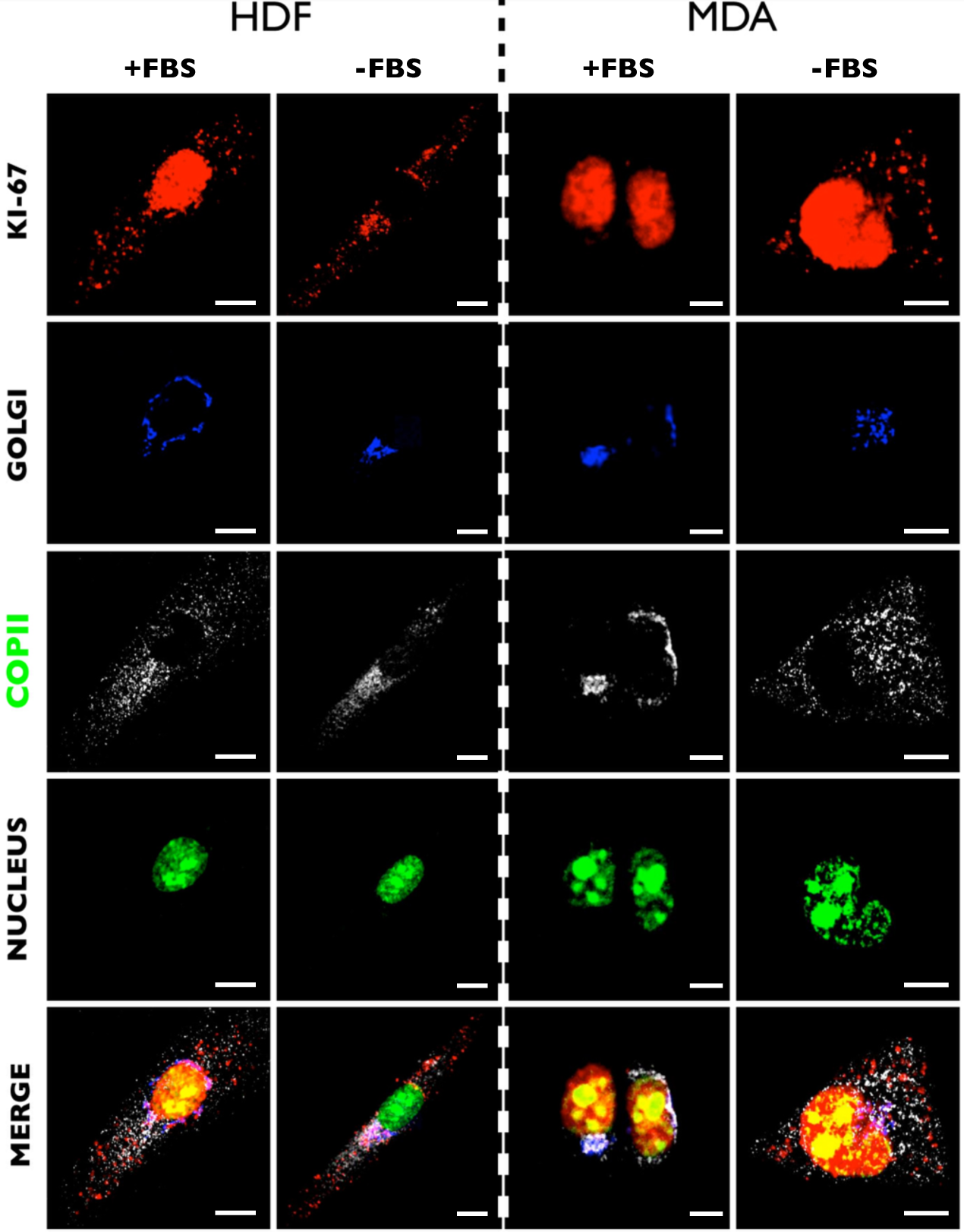


**Fig G.** **Confocal analyses showing the co-localisation between COPII and KI-67.** Here we show all the different channels split each other from figure 6 in the main article for a better visualisation.


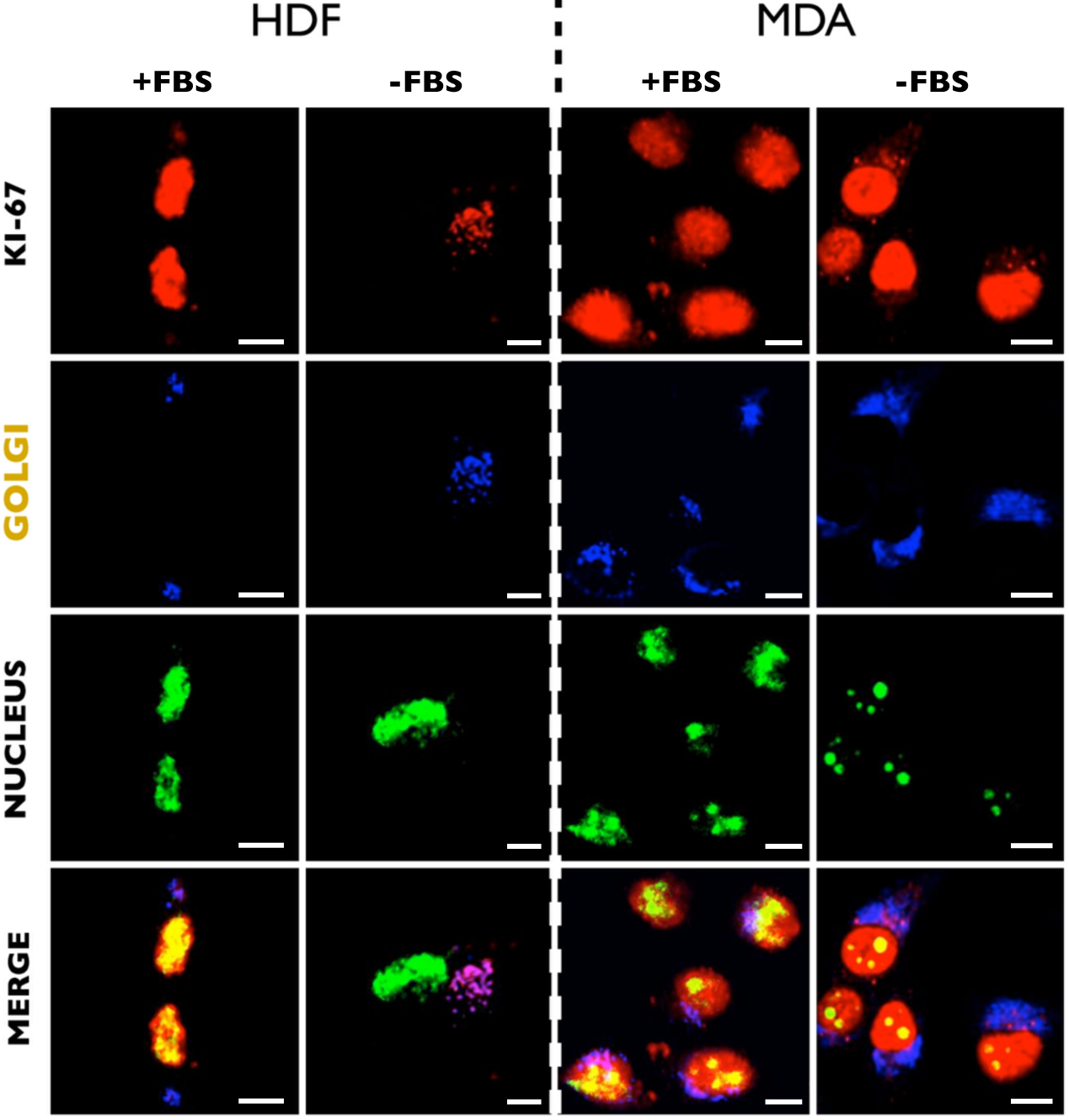


**Fig H. Confocal analyses showing the co-localisation between the Golgi apparatus and KI-67.** Here we show all the different channels split each other from figure 6 in the main article for a better visualisation.
